# Deleterious impact of COVID-19 pandemic: Male fertility was not out of the bag

**DOI:** 10.1371/journal.pone.0284489

**Published:** 2023-05-08

**Authors:** Siwar Garrouch, Amira Sallem, Manel Ben Fredj, Rim Kooli, Manel Bousabbeh, Ines Boughzala, Asma Sriha, Awatef Hajjaji, Meriem Mehdi

**Affiliations:** 1 Laboratory of Cytogenetics and Reproductive Biology, Maternity and Neonatology Center, Fattouma Bourguiba University Teaching Hospital, Monastir, Tunisia; 2 Laboratory of Histology-Embryology and Cytogenetics (LR18ES40), Faculty of Medicine of Monastir, University of Monastir, Monastir, Tunisia; 3 Department of Epidemiology and Preventive Medicine, Fattouma Bouguiba University Hospital, University of Monastir, Monastir, Tunisia; 4 Department of Gynecology and Obstetrics, Maternity and Neonatology Center, Fattouma Bourguiba University Teaching Hospital, Monastir, Tunisia; American University of Madaba, JORDAN

## Abstract

**Background:**

The emergence and the spread of coronavirus disease (COVID-19) induced by the SARS-CoV-2 virus has multiple consequences in all countries around the world. Male germ cells of infertile patients which are shown to be vulnerable to many environmental conditions, could be particularly vulnerable to such an exceptional pandemic situation. We aimed through the current study to investigate the potential variations in sperm quality of infertile patients during the COVID-19 pandemic in Tunisia.

**Methods:**

This was a cohort study including 90 infertile patients addressed to Laboratory of Cytogenetics and Reproductive Biology of Monastir Department of Maternity and Neonatology in Monastir, during the two first COVID-19 waves in Tunisia and who already have a spermogram before the pandemic period.

**Results:**

We have pointed out a significant decrease in both total and progressive sperm motility during COVID-19 pandemic (p<0.0001 and p = 0.001 respectively). The percentage of morphologically abnormal spermatozoa increased from 90.99±7.38 to 93.67±4.55% during the pandemic (p< 0.001). The remaining sperm parameters were similar between the two compared timepoints. Interestingly, the univariate analysis didn’t show any other associated factor to the observed impairment in sperm mobility and morphology.

**Conclusion:**

These data highlight the severe impact of the pandemic of the male reproductive health of hypofertile patients. Delaying infertility investigations and management after pandemic waves is recommended to hope a better gamete quality and hence to improve conception potential.

## Introduction

In December 2019, the severe acute respiratory syndrome coronavirus 2 namedSARS-CoV-2 and caused by the SARS-CoV-2 virus belonging to the family *Coronaviridea* has appeared as a sudden spike of illness in Wuhan, China. This outbreak was not contained, it spreads across the globe to touch over 222 countries and territories [1]. The pandemic character of COVID‐19 has been declared by the World Health Organization (WHO) on March 11^th^, 2020. Since the appearance of first cases until April 2022, the WHO has recorded more than 500 million of cases resulting in over 6 millions of deaths [2]. In Tunisia, The COVID-19 pandemic officially started on March 2, 2020. On May 5^th^, 2022, the number of deaths reached 28566 and all 24 governorates were affected [3]. The respiratory tract was initially described as the main target of the SARS-CoV-2 but the virus was shown later to trigger tissues alterations in multiple organs belonging to the gastrointestinal tract [4], the pulmonary system [5], the cardiovascular system and other organs [6]. The virus invades the host cell by fixing to ACE2 (*Angiotensin*-*Converting Enzyme 2)* receptor. It was hence expected that tissues and organs cells expressing ACE2 receptor could be possible targets of the SARS-CoV-2 [7]. As the ACE2 receptor was shown to be highly expressed by several cell types in the testes [6], the question to answer was whether there was any impairment in semen quality in COVID-19 patients and particularly those being infertile. Infertility is defined by The WHO as the inability to conceive after a period of at least 12 months of regular unprotected sexual intercourse. It is considered as a public health issue concerning between 48 and 186 million couples around the world [8]. Due to their inability to conceive, infertile patients are daily facing a familial and social pressure lowering their self-esteem [9]. Such a critical psychological status could be worsened in a pandemic context. Furthermore, in such an exceptional pandemic context, the sexual life of infertile couples could be disrupted as the possibility of sexual transmission of the virus could not be completely discarded mainly because the lack of scientific proofs on the topic [6]. Such considerations raise the possibility that infertile patients who are basically more vulnerable to distress, could be particularly impacted by the COVID-19 pandemic in terms of gametes quality even in the absence of biological proof of SARS-CoV-2 infection. Given the absence of data on the reproductive health of Tunisian infertile patients during COVID-19 pandemic, the current study was conceptualized to investigate the potential impact of that particular context on the semen quality of infertile men.

## Methods

### Study design

This was a cohort study performed in the Laboratory of Cytogenetics and Reproductive Biology of Monastir (Maternity and Neonatology Center, Monastir).

### Study participants

We included in the current study male patients who were addressed to the laboratory for sperm analysis during the first or the second COVID-19 wave in Tunisia and who already have a spermogram before the pandemic period. Hence, two time points were considered: T1: before the COVID-19 pandemic and T2: during the COVID-19 pandemic. For patients having more than one spermogram before the COVID-19 pandemic, the newest was considered. During the pandemic period, patients were not asked to show proof of negative COVID-19 PCR (polymerase chain reaction) because none of our patients had COVID-19 symptoms and according to the rules in that period PCR was not indicated. Spermogram appointment was postponed if the patient had any of the COVID-19 symptoms. We didn’t include patients who received medication (antibiotics, antivirals, anti-inflammatory, antioxidants) between the two compared spermograms. We didn’t also include patients who had undergone urogenital surgery (for varicocele, inguinal hernia) during that period.

### Semen parameters assessment

Semen samples were obtained by masturbation in the laboratory after 3–5 days of sexual abstinence. Semen analysis was carried out by a trained technician at the Laboratory of Cytogenetics and Reproductive Biology (Monastir, Tunisia). Sperm analysis as well as results interpretation were performed according to 2021 WHO guidelines [10]. Semen parameters analysis is performed 30 min after ejaculation to allow sperm liquefaction. It includes a macroscopic (sperm volume, viscosity and pH) and a microscopic examination (sperm motility, vitality, concentration and morphology). To assess sperm morphological abnormalities the modified DAVID classification (Tian, Li, Zhang, & Xu, 2016) was used. Sperm morphology is evaluated for 100 spermatozoa and expressed as the percentage of morphologically abnormal spermatozoa. The Multiple Abnormalities Index (MAI) is calculated according to the following formula:

MAI=Totalnumberofdetectedabnormalities/Totalnumberofmorphologicallyabnormalspermatozoa.

### Ethical considerations

The current study was approved by the Ethics Committee of the Faculty of Medicine of Monastir under the number IORG 0009738 N°115/ OMB 0990–0279. All patients have provided written informed consent. During the study period, we carefully protected the privacy and the confidentiality of personal informations of the included patients. Only the investigator knows patients’ identities. A code number was assigned to each patient and the list linking names to the codes being kept separately in a secure place, with a limited access. Only the investigator was able to link trial data back to specific individuals.

### Statistical analysis

Statistical analysis was performed using Statistical Package for Social Sciences (SPSS) version 23.0. Data were represented as mean±standard deviations (SD) or median and quartiles for quantitative variables and as number (n) and percentages (%) for qualitative variables. In order to compare semen parameters before and during the COVID-19 pandemic, we conducted a univariate analysis using the matched samples Student t-test and Wilcoxon test for quantitative variable with symmetric distribution and asymmetric distribution, respectively. We compared the sample distribution by kolmogrov-Simrnov test (K-S). We used MCnemar test matched samples when comparing qualitative variables. Univariate analysis was performed using Mann Whitney test, Khi square test and t-test for independent samples. A p value<0.05 were considered statistically significant.

## Results

### Sociodemographic and clinical characteristics of the study population

The current study included a total of 90 patients aged between 28 and 70 years old with a mean age of 38.81±7.69 years old. The predominant age range is that between 30 and 39 years old (55.1%). Most of participants were living in urban areas (83.3%). All participants were active workers among whom48.9%having professions that could potentially compromise fertility.

When focusing on personal history, 25% of our patients had medical history, 20.5% had a chirurgical history and 18.2% had urogenital history (varicocele, testicular ectopia, inguinal hernia, urogenital infection, testicular trauma with or without testicular torsion). On the other hand, family infertility history was found among 9.1% of the participants. Primary infertility was the most common (75%) in the studied population. After investigating the possible causes of couple infertility, male factors were identified in 66% of cases. For the remaining cases both male and female infertility factors were implicated (34%). The median infertility duration was of 8 months with extremes varying from 2 to 20 months. At physical examination, Body Mass Index (BMI) was normal in 40.6% of the study population, while overwaited and obese patients represented 43.2% and 16.2% respectively. With regards to lifestyle factors, approximately 62% of our patients were active smokers and 26.4% drink alcohol.

### Comparison of Semen parameters before and during COVID-19 pandemic

During the two considered waves of the pandemic, the absenteeism rate for spermograms appointment was of 33%. The median value of the delay between the two compared spermograms (T1 and T2) was of 9.50 [3.75–23] months. The mean sexual abstinence delay was of 3.19 ±0.56days for pre-COVID-19 spermograms and of 3.17 ±0.59days for pandemic-spermograms (p = 0.787). Comparisons between the different semen parameters at the two compared timepoints (T1 et T2) are detailed below.

#### Quantitative semen parameters

No significant difference was noticed before and during the pandemic period with regards to semen volume, semen pH, sperm viability and sperm count(p = 0.686; p = 0.877; p = 0.136 and p = 0.079 respectively).

The median value of total as well as progressive sperm motility was significantly decreased during COVID-19 wave (p<0.0001 and p = 0.001 respectively). The percentage of morphologically abnormal spermatozoa rose from 90.99±7.38 (%) before the pandemic to reach 93.67±4.55 (%) during it (p<0.001). Teratozoospermia was observed in 87.2% of cases before the pandemic compared to 96.1% during it.

Abnormalities association as assessed by the MAI (Multiple Abnormalities Index) was similar between the two compared periods (p = 0.493). This latter was pathological in 89.5% before COVID-19 vs 85.3% during the pandemic. Comparisons of semen parameters between the two periods are shown in Table 1 for variables which were normally distributed and in Table 2 for those which were asymmetrically distributed.

**Table 1:** Comparison of normally distributed quantitative semen parameters before and during COVID-19 pandemic.

Variables	Before COVID-19 pandemic	During COVID-19 pandemic	Mean of differences ± SD of the mean of differences	p-value
**Semen volume (mean±SD)**	3.20±1.85	3.18±1.98	0.07 ±1.64	0.686
Normal n(%)	70(83.3)	76(87.4)		
Pathologicaln(%)	14(16.7)	11(12.6)		
**Semen pH**	7.92±0.19	7.92±0.19	-0.05±0.26	0.877
Normal n(%)	59(76.6)	55(73.3)		
Pathologicaln(%)	18(23.4)	20(26.7)		
**Multiple Abnormalities Index**	1.85±0.28	1.83±0.26	-0.02±0 .27	0.493
Normal n(%)	8(10.5)	11(14.7)		
Pathologicaln(%)	68(89.5)	64(85.3)		
**Spermmorphology**	90.99±7.38	93.67±4.55	2.31±15.73	<0.000
Normal n(%)	10(12.8)	3(3.9)		
Pathologicaln(%)	68(87.2)	73(96.1)		

**Table 2:** Comparison of asymmetrically distributed quantitative semen parameters before and during COVID-19 pandemic.

Variables	BeforeCOVID-19 pandemic	During COVID-19 pandemic	p-value
**Sperm count (Million/ml) Median [IQR** ^*^ **]**	52 [16.3–119.5]	60[10.3–128]	0.079
Normal n(%)	68(76.4)	65(73.0)	
Pathological n(%)	21(23.6)	24(27.0)	
**Total sperm motility** **Median [IQR** ^*^ **]**	40[30–45]	35[21.2–40]	<0.000
Normal n(%)	51(69.9)	12(16.7)	
Pathologicaln(%)	22(30.1)	60(83.3)	
**Progressive sperm motility Median [IQR** ^*^ **]**	20[15–25]	20[10–25]	0.001
Normal n(%)	17(23.3)	8(11.1)	
Pathologicaln(%)	56(76.7)	64(88.9)	
**Leukocytes(Million/ml) Median [IQR** ^*^ **]**	0.30[0.10–1.10]	0.20[0.08–0.62]	0.129
Normal n(%)	60(72.3)	69(81.2)	
Pathologicaln(%)	23(27.7)	16(18.8)	

#### Qualitative semen parameters

The difference between the two values didn’t reach statistical significance in both sperm agglutination and sperm viscosity (p = 0.250; p = 0.727 respectively).

### Associated factors of sperm motility and morphology impairment during COVID-19 pandemic (univariate analysis)

We compared sociodemographic and clinical characteristics between patients whose sperm morphology and motility were aggravated during the pandemic to those who have improved or stable values during the pandemic. Sociodemographic and clinical characteristics were similar among patients with altered sperm motility and morphology compared to those with stable or improved sperm motility and morphology during COVID-19 pandemic as shown in Tables 3–5.

**Table 3:** Comparison of sociodemographic and clinical features between the two groups with regards to initial total sperm motility impairment during COVID-19 pandemic.

Variables	Group 1’	Group2’	P value
**Age : mean±SD**	37.5±6.7	40.9±9.2	0.086
**BMI : mean±SD**	25.8±3.59	27.4±4.27	0.335
Infertility duration Median[IQR^*^]	16[9.5–36]	14[8–36]	0.426
**Infertility type : n(%)**			0.232
Primary	33(80.5)	19(67.9)	
Secondary	8(19.5)	9(32.1)	
**Infertility origin: n(%)**			0.488
Male	26(63.4)	20(71.4)	
Mixed	15(36.6)	8(28.6)	
**Tobacco users: n(%)**	24(61.5)	16(57.1)	0.718
**Alcoholconsomers : n(%)**	9(23.1)	8(28.6)	0.610
**Family history of infertility: n(%)**	3(7.3)	3(11.1)	0.675
**Comorbidities :n(%)**			
Medical	12(29.3)	4(14.8)	0.169
Surgical	10(24.4)	3(11.1)	0.173
Urogenital	8(19.5)	7(25.9)	0.533
**Professional exposure to reprotoxicenvironment : n(%)**	26(63.4)	11(39.3)	0.048

Group1’: patients with total sperm motility impairment during the COVID-19 pandemic; Group 2’: patients without total sperm motility impairment during the COVID-19 pandemic

**Table 4:** Comparison of sociodemographic and clinical features between the two groups with regards to initial progressive sperm motility impairment during COVID-19 pandemic.

Variables	Group1”	Group2”	p-value
**Age : mean±SD**	38.8±7.6	39±8.4	0.929
**BMI : mean±SD**	25.64±3.91	27.50±3.90	0.238
Infertility duration Median[IQR^*^]	12[7.75–25]	24[10.5–40]	0.180
**Infertility type : n(%)**			0.728
Primary	27(77.1)	25(73.5)	
Secondary	8(22.9)	9(26.5)	
**Infertility origin:n(%)**			0.865
Male	23(65.7)	23(67.6)	
Mixed	12(34.3)	11(32.4)	
**Tobacco users: n(%)**	21(63.6)	19(55.9)	0.518
**Alcohol consumers: n(%)**	9(27.3)	8(23.5)	0.725
**Family history of infertility: n(%)**	2(5.9)	4(11.8)	0.673
**Comorbidities : n(%)**			
Medical	11(31.4)	5(15.2)	0.114
Surgical	8(22.9)	5(15.2)	0.419
Urogenital	9(25.7)	6(18.2)	0.454
**Exposed profession : n(%)**	19(54.3)	18(52.9)	0.911

Group 1”: patients with progressive sperm motility impairment during the COVID-19 pandemic; Group 2”: patients without progressive sperm motility impairment during the COVID-19 pandemic

**Table 5:** Comparison of sociodemographic and clinical features between the two groups with regards to sperm morphology impairment during COVID-19 pandemic.

Variables	Group A	Group B	p-value
**Age : mean±SD**	38.7±7.7	38.8±8.45	0.982
**BMI : mean±SD**	26.6±4.8	26.25±3.76	0.842
Infertility duration Median[IQR^*^]	24[11–36]	14[9–36]	0.546
**Infertility type :n (%)**			0.592
Primary	15(71.4)	41(77.4)	
Secondary	6(28.6)	12(22.6)	
**Infertility origin: n (%)**			0.275
Male	12(57.1)	38(70.4)	
Mixed	9(42.9)	16(29.6)	
**Tobacco users:n (%)**	16(76.2)	30(57.7)	0.138
**Alcohol consumers: n (%)**	8(38.1)	12(23.1)	0.193
**Family history of infertility: n (%)**	2(9.5)	4(7.5)	1
**Comorbidities :n (%)**			
Medical	6(28.6)	13(24.5)	0.720
Surgical	3(14.3)	12(22.6)	0.532
Urogenital	8(38.1)	7(13.2)	0.25
**Exposed profession : n (%)**	14(66.7)	25(46.3)	0.113

Group A: patients with sperm morphology impairment during the COVID-19 pandemic; Group B: patients without sperm morphology impairment during the COVID-19 pandemic

## Discussion

COVID-19 pandemic is exceptional worldwide health situation which still have devastating medical, economic, and sociocultural impacts. The most commonly described infection was that of the respiratory system. However, multiple organs were shown to be possible targets for the SARS-CoV-2 virus [11]. Several studies have focused on the impact of COVID-19 in numerous human’s functions [12–16], but data illustrating the impact of SARS-CoV-2 on the urogenital male tract still to be scarce [17, 18] and those evaluating the impact of the pandemic context on the reproductive health are so limited [19, 20]. The current study is, to the best of our knowledge, the first Tunisian and Nord African study to investigate male reproductive health through semen parameters analysis during the two first waves of the COVID-19 pandemic. During this historical cohort study, we collected baseline data related to 90 patients that attended the laboratory before and during COVID-19 pandemic. None of them had a proof of a positive PCR (polymerase chain reaction) test or COVID-19 symptoms. The results of the current study highlighted the impairment in sperm quality during the pandemic as two main sperm parameters were shown to be altered: sperm motility (both total and progressive) and morphology. When focusing on the sociodemographic and lifestyle characteristics of the study population, we mainly noticed that almost half of the attendees (48.90%) have a profession that could compromise fertility and that tobacco consumption was noticed in more than half of the study population (62%). Patients were more likely to have overweight and obesity than a normal BMI. Although investigating the effects of sociodemographic and lifestyle factors was not one of the aims of the current study, it is important to note that high percentages of tobacco consumption, professional exposure to reprotoxic environment and obesity as noticed in the study population are known to have deleterious impact on semen quality [21–24]. However, as we didn’t include any patient who have a modification in any of these features between the two considered time points (T1 and T2), the observed alteration in semen quality isn’t related to variation in these parameters. With regards to infertility characteristics, primary infertility was the most common (75%) and male infertility factors were identified in 66.6% of the participants to the current study. Indeed, when focusing on the baseline semen parameters (before the pandemic), we noticed an alteration of many semen parameters as compared to the WHO 2021 thresholds. Both total and progressive sperm motility were decreased in more than 75% of the study population. A decrease below the WHO thresholds was also seen in sperm morphology (78.20% of cases) and MAI (89.50% of cases).This predominance of male infertility related factors was also reported in other studies [25, 26] and could be explained by the observed high percentages of sociodemographic and lifestyle factors (tobacco, occupation exposure and obesity) shown to be a threat for the male reproductive health. The reproductive health especially the male one could be compromised by a multitude of factors. Spermatozoa are shown to be vulnerable to many intrinsic as well as extrinsic conditions. Sperm motility and morphology which were significantly impaired during COVID-19 pandemic in the current study are two crucial parameters in the fertilizing potential during spontaneous and medically assisted conceptions [27, 28]. Mainly two hypotheses could be advanced to explain the decline in these two parameters: (i) the recruited patients are infected by the SARS-CoV-2 virus when performing the second spermogram, (ii) the stressful pandemic context has affected spermatogenesis course. To address the first hypothesis, it is important to mention that patients who were addressed to the laboratory during COVID-19 waves were not asked to show a proof of negative SARS-CoV-2 PCR. However, those with any of COVID-19 symptoms were asked to postpone sperm analysis after recovery. This makes this hypothesis implausible but could not be totally discarded due to the lack of biological proof. Based on previous studies, semen parameters mainly semen volume, sperm count, motility and morphology are shown to be influenced by viral infections such as HIV (Human Immunodeficiency Virus), HHV (Human Herpes Virus), HBV (Hepatitis B Virus), Ebola, mumps, and Zika viruses [29]. The impact of SARS-CoV-2 infection on semen parameters has still to be unraveled. Indeed, several studies [29–33] have pointed out a significant impairment in many semen features including motility and morphology, contrasting to others who have concluded that there was no impact of COVID-19 infection on semen parameters [34].The potential impact of SARS-CoV-2 virus on the reproductive function could be mainly mediated by two possible pathways: (i) a target cells invasion by the SARS-CoV-2 virus, (ii) a SARS-CoV-2 induced inflammatory response in the reproductive tract. SARS-CoV-2 enters into target cells by engaging the ACE-2 (Angiotensin-Converting Enzyme 2) receptor and employing the transmembrane protease serine type 2(TMPRSS2) for S protein priming [35]. Hence, to address this topic, the first question to be answered is whether the SARS-CoV-2 is expressed in the reproductive tract or not. The transcriptome sequencing results published by Wang in 2020 have confirmed that the reproductive system is a target of SARS-CoV-2 as ACE receptor coding gene was found to be expressed at the membrane of Sertoli and Leydig cells as well as spermatogonia [36]. Moreover, they demonstrated that genes implicated in the spermatogenesis process and those encoding for mitochondrial functions that are crucial for sperm motility were compromised in ACE-2 positive Leydig cells, Sertoli cells and spermatogonia. ACE-2 positive germ cells were also characterized by a low expression of genes involved in sperm chromatin condensation, meiosis, capacitation, acrosome reaction and sperm-oocyte recognition. These findings highlighted the harmful effect of SARS-CoV-2 virus not only on spermatogenesis but also on the different steps of the fertilization process. Holtmann and collaborators [29] were the first to reveal the deleterious impact of SARS-CoV-2 infection on semen parameters. They included 18 men with COVID-19 symptoms and 14 healthy control men. The authors established a considerable decrease in sperm count and both total and progressive motility in COVID-19 positive men as compared to their healthy counterparts. Impairment in sperm motility was also described by Ma and collaborators in 4 patients out of 12 (33.30%) infected by the SARS-CoV-2 [37]. The study performed by Maleki [32] and comparing semen parameters between a cohort of 84 laboratory-confirmed COVID-19 patients to 105 healthy men has shown a significant decrease in semen volume, sperm concentration and morphology. Interestingly, the observed alterations were correlated to the seminal plasma ACE2 enzymatic activity as well as to the disease severity. Testicular ACE2 expression was proven to be age related [38]. The highest level of ACE2 mRNA was recorded in patients in their thirties and the lowest among 60 years-old patients. The above-mentioned findings might provide evidence that young patients are at higher risk of testicular damage than older ones. This theory could match with our results as the mean age of our study population was of 38.81±7.69 years old. Once again, this interpretation should be cautiously considered as we have no biological proof of COVID-19 infection in the current study. As data available so far are not robust enough to draw firm conclusion with regards to the impact of SARS-CoV-2 on semen quality, some researchers have investigated whether the SARS-CoV-2 RNA is present in the semen of positive COVID-19 patients. The two first published studies on the topic were that of Li and collaborators [30] and Pan and colleagues [39]. In the first study, SARS-CoV-2 RNA was found in 6 semen samples out of 38 (15.80%). RNA positive semen samples belong to 4 patients in the acute stage and 2 recovered patients. However, in the second study, none of the 34 analyzed samples were positive for the SARS-CoV-2 RNA. In the latter study, samples were collected after 1 month from COVID-19 diagnosis even in patients (6 out of 34; 19%) who reported scrotal discomfort suggesting SARS-CoV-2 orchitis during the acute stage of the disease. These data were later corroborated by cohort studies which included a total of 342 semen samples belonging to 63 patients in the acute stage and 279 recovered ones [29, 40–46]. None of these studies has demonstrated the presence of viral RNA in the semen. Taken together, these findings support the hypothesis of a possible contamination in the six semen samples where the viral RNA was found. This question was addressed at the end of 2021 by Delaroche and colleagues [47] who have firstly detected SARS-CoV-2 RNA in one patient among 32 COVID-19 volunteers and then have demonstrated the contamination of the SARS-CoV-2 positive semen specimen by oropharyngeal bacteria suggesting a non-respect of strict aseptic recommendations during sperm collection. Moreover, as the viral culture was negative, the hypothesis of oral or manual contamination during semen collection seems the most plausible. The absence of viral RNA in the semen supports the second hypothesis stipulating that impairment of semen quality in SARS-CoV-2 patients is possibly related to the virus-induced inflammatory response in the reproductive tract. SARS-CoV-2 infection induces a cytokine storm also called cytokine-release syndrome (CRS) corresponding to a dysregulated and excessive production of early response pro-inflammatory cytokines [48]. This storm is associated with higher release of interleukin-6 (IL-6), IL-7, IL-8 and alpha tumor necrosis factor (TNF-α) by leukocytes, macrophages and T cells. These elements are shown to act as proapoptotic factors during inflammatory process in the testis [49]. Moreover, leukocytes and immune cells can pass the blood-testis barrier and produce interferons dysregulating steroidogenesis and inhibiting testosterone production by Leydig cells [50]. Additionally, COVID-19 positive patients may have hyperthermia or even fever which could have a deleterious impact on the spermatogenesis course and explain the observed alterations in semen parameters [51]. Indeed, Patel and colleagues have shown an impairment in both sperm count and motility in participants with verified COVID-19-associated fever [52]. The second hypothesis that we advanced to explain the decrease in sperm motility and the increase in morphologically abnormal spermatozoa as shown in the current study, was the stress induced by the pandemic context. Outside of the pandemic context, infertile men are subject to greater stress and are vulnerable to psychological disorders compared to healthy men. Couples with infertility issues are daily facing a social pressure which may cause further distress and lower self-esteem. These feelings could be aggravated by the financial difficulties related to the cost of infertility investigation tests as well as the cost of ART (Assisted Reproductive Technologies) procedures [53].The high rate of absenteeism for spermograms appointments (33%) during the two fist waves of the disease in Tunisia as shown in the current study, could be an indirect marker of the pandemic related anxiety in infertile patients who despite the need to investigate infertility issues, have been reluctant to attend the laboratory may be to avoid the possible risk of contamination. When examining psychological reactions during the emergence and the first wave of SARS-CoV-2 infection in Chinese people, many features mainly related to anxiety, depression, sleep disturbances, and suicide attempts were pointed out even in patients with no preexisting psychiatric disorders [54]. People were worried about the high transmissibility of the infection. Hence, sexual desire and frequency were shown to be decreased in both genders and a significant decrease in fertility performance was reported during such a period of psychological crisis [55]. Among COVID-19 survivors, erectile dysfunction was described as a consequence of endothelial dysfunction leading to reproductive health disturbances [56]. Moreover, chronic exposure to such psychological distress might lead to permanent stimulation of the hypothalamic–pituitary–adrenal (HPA) axis which in turns could inhibit the reproductive function. In accordance with the results of the current study, infertile patients were shown to exhibit an alteration in semen quality mainly with regards to sperm count, motility and nuclear sperm DNA quality [55].In the current study the link between distress and the observed impairment in semen parameters was one of the main hypotheses that we advanced. However, we don’t have a quantitative evaluation of the hypothesized stress. Before the pandemic period, a study on the anxiety and depression levels among infertile patients was conducted in the Laboratory of Cytogenetics and Reproductive Biology of Monastir. Since the beginning of the first wave of COVID-19 in Tunisia, the medical staff interrupted the study. Indeed, the research was consisting of administering the HAD (Hospital Anxiety and depression) scale to the patients to assess the level of anxiety and depression on the day of semen analysis. As one of the restrictive recommendations in managing patients during the pandemic was to shorten the human contact delay, the study was suspended. The ATME (Tunisian Association of Embryologists) and the STGO (Tunisian Society of Gynecology and Obstetrics) have established a list of recommendations to better guide ART practitioners in managing infertility issues during the crisis. These measures of distancing, limiting the number of patients in the laboratory and disinfecting may increase the anxiety of patients on the day of semen collection. Psychological stress is demonstrated to be alone a source of sperm quality degradation related to a significant decrease in seminal antioxidants such as glutathione (GSH) and free sulphydryl [57]. In accordance with the results of the current study, the two impaired sperm parameters in the latter study were motility and morphology in patients exposed to high levels of stress. Depletion in seminal antioxidants during stress conditions could be deleterious to spermatozoa as reactive oxygen species (ROS) could attack cell membrane phospholipids and induce lipid peroxidation [57]. Aiming to determine possible associated factors to the noticed impairment in sperm motility and morphology, we performed the univariate analysis. We have interestingly shown that among sociodemographic and clinical features there were no associated factors with sperm quality alteration except the pandemic context. These data reinforce the association between the pandemic circumstances and the drop of semen quality in hypofertile patients. This was at the best of our knowledge, the first Tunisian study to investigate semen quality variation during first two waves of COVID-19 in Tunisia. Interestingly, semen parameters of the same patients were evaluated before and during the pandemic so that we limited possible interindividual variations and hence bias. The significant decrease in sperm motility (both progressive and total) 30 minutes and as well as the increase in the percentage of morphologically abnormal spermatozoa highlighted the deleterious impact of pandemic context on infertile Tunisian patients even with no biological proof of SARS-COV-2 acute infection. Sperm motility and morphology are crucial for the success of fertilization during spontaneous and assisted conception. Hence, the observed alterations could be a real threat for the occurrence of conception in childless couples. It bears noting that our study has some limitations mainly related to the lack of quantitative evaluation of the level of perceived stress among infertile patients and whether it’s correlated to the decrease in semen quality. The decision of the medical staff in our laboratory was to prioritize patients’ safety and to interrupt any research which could extend the duration of the patient’s stay in the lab. Moreover, it will be of great interest to explore seminal oxidative stress to know whether the observed decline in sperm motility and morphology is the mirror of an imbalance between seminal antioxidant and ROS generation in such a pandemic context. Reassessing the semen parameters of the included patients away from COVID-19 waves could be also of great interest to check the reversibility of the observed alterations

## Conclusion

The context of COVID-19 pandemic is a particular environmental condition which placed peoples under particularly stressful circumstances. Infertile patients are more likely to be vulnerable to such a stress. The decision of postponing the appointments of non-urgent ART procedures and infertility investigations was announced by the Tunisian Association of Embryologists (ATME) and the Tunisian Society of Gynecologists and Obstetricians (STGO) in April 2020 and maintained for almost 2 months. Outside of that period, infertile patients were received in andrology laboratory for sperm analysis and only those who reported having COVID-19 related symptoms were asked to postpone their appointment. For the remaining patients, no biological test confirming the absence of COVID-19 infection was required. The results of the current study highlighted the impairment in sperm quality during the pandemic as two main sperm parameters were shown to be altered: sperm motility (both total and progressive) and morphology. The observed alterations are relevant to point out because they affect two crucial sperm parameters during the fertilization process in both natural and assisted conceptions. Whether related to stressful pandemic conditions or non-diagnosed SARS-CoV-2 infection, these data support the vulnerability of male gametes. According to our results, delaying infertility investigation and treatment after the pandemic could improve gametes quality and hence pregnancy rate in couples seeking for fertility.

## Supporting information

S1 Dataset (SAV)
